# Knowledge Performance, User Experience, and Cybersickness With Immersive Virtual Reality for Tai Chi Movement-Encoding in Older Adults: Randomized Comparison Study

**DOI:** 10.2196/96867

**Published:** 2026-09-30

**Authors:** XiaCheng Song, Lu Sun, Yaqing Song, Huafeng Qu, Jing Jin, Junfeng Zhu, Wenwen Song, Huirong Huang

**Affiliations:** 1 Guangdong University of Technology, Guangzhou, Guangdong, China; 2 Xi'an Fanyi University, Taiyigong, Chang'an District, Xi'an, Shaanxi, 710105, China, 86 18717371790; 3 Shaanxi Technical College of Finance & Economics, Xi'an, Shaanxi, China; 4 Yunnan College of Business Management, Kunming, Yunnan, China; 5 Guangdong University of Science and Technology, Dongguan, Guangdong, China; 6 Zhaoqing University, Zhaoqing, Guangdong, China; 7 Xidian University, Xi'an, Shaanxi, China; 8 Guangdong Industry Polytechnic, Guangzhou, Guangdong, China

**Keywords:** human factors, ergonomics, immersive virtual reality, older adults, movement encoding, cybersickness, VRSQ, Virtual Reality Sickness Questionnaire

## Abstract

**Background:**

Immersive virtual reality (VR) is increasingly used for embodied training, but its practical value for older adults depends on whether the experiential benefits can be achieved without unacceptable discomfort or overstated claims about motor learning.

**Objective:**

This randomized study compared head-mounted immersive VR with standardized, content-matched 2D video instruction for Tai Chi movement-encoding in community-dwelling older adults.

**Methods:**

A total of 106 older adults were randomized to immersive VR (n=56) or video instruction (n=50). The primary analysis included 92 participants with complete age, pretest, and posttest data (VR: n=45; Video: n=47). The primary outcome was a 5-item movement-encoding knowledge score (range 0‐20), analyzed using analysis of covariance, with posttest score as the dependent variable and group, pretest score, and age as predictors. Secondary outcomes were intrinsic motivation, game experience, and learning engagement; safety, tolerability, and feasibility were assessed using the Intrinsic Motivation Inventory, Game Experience Questionnaire, Utrecht Work Engagement Scale–Short Form, participant flow, and Virtual Reality Sickness Questionnaire. VR system telemetry was analyzed descriptively as an exploratory process outcome.

**Results:**

Adjusted posttest movement-encoding performance did not differ between VR and video instruction (adjusted difference −0.27, 95% CI −2.25 to 1.72; *P*=.79; partial η^2^=0.001). The Game Experience Questionnaire results favored VR for competence (Video: mean 3.36, SD 1.21; VR: mean 4.15, SD 0.92; *q*=0.009), flow (Video: mean 3.77, SD 1.26; VR: mean 4.46, SD 0.76; *q*=0.014), and lower tension (Video: mean 1.76, SD 1.04; VR: mean 1.27, SD 0.63; *q*=0.037), whereas challenge did not remain significant after false discovery rate correction (*q*=0.073). In the VR Group, recognition accuracy was associated with posttest movement-encoding score (n=44; *r*=0.317, *P*=.04), but pose accuracy was not (n=44; *r*=0.129, *P*=.41). The Virtual Reality Sickness Questionnaire scores were low among valid VR participants (total score mean 3.77, SD 11.12), and 36 out of 46 (78%) participants reported zero symptoms.

**Conclusions:**

With a strong active control, immersive VR did not produce higher short-term Tai Chi movement-encoding performance than video instruction. Among participants who completed the VR session, the immediate game-experience profile was more favorable. These findings indicate that VR may offer experiential benefits, while also highlighting the need to manage cybersickness and technical exclusions and to distinguish movement knowledge from actual motor execution.

## Introduction

### Rationale for the Study

As societies age, maintaining functional independence has become a central public health priority, particularly given the heightened consequences of balance failure and mobility decline in later life [1-3]. Tai Chi is frequently recommended for older adults because it combines controlled postural transitions with sustained attention to movement elements and goal-directed practice that can be adapted to heterogeneous functional levels [4-6]. Unlike purely verbal or screen-based cognitive tasks, Tai Chi learning requires embodied visuomotor mapping and multisensory integration, making it a suitable test bed for studying how immersive virtual reality (VR) shapes action-related memory encoding in later life [7-9]. Yet, the real-world dissemination of Tai Chi in community contexts faces a persistent accessibility paradox: the individuals who could benefit most from structured practice often have the least reliable access to safe, repeatable instruction [10-12]. Shortages of qualified instructors, geographic and scheduling constraints, and the practical challenges of maintaining consistent supervision can all limit participation [11,13,14]. Additionally, complex movement learning may carry a psychological barrier for novice learners, as uncertainty about “doing it correctly” can discourage sustained engagement even when motivation to improve health is high [11,13,15].

Immersive VR has been proposed as a scalable approach to deliver standardized movement learning experiences under controlled conditions, offering a headset-based practice space that can reduce external distraction and support repeated exposure to the same instructional sequence [10,16,17]. However, the evidence base for VR and related immersive training and learning, especially for de novo acquisition of complex movement sequences, remains fragmented and often inconsistent when compared with strong active controls [18-20]. Recent randomized studies and reviews likewise suggest that outcomes depend on training dose, adaptive design, comparator strength, and the distinction between device-recorded performance and transferable motor learning [21-24]. A key methodological limitation is that many studies rely on passive or no-treatment controls, making it difficult to isolate the effect of immersion from instructional content and practice structure. This concern echoes longstanding debates in media-comparison research: learning gains are often driven by instructional design and practice opportunities rather than the delivery medium per se [25,26]. For older adults, these issues are further complicated by immersion-specific feasibility and safety constraints, including VR discomfort and symptoms that may affect adherence and threaten generalizability if not transparently measured and reported [24,27-30].

Beyond these methodological challenges, 2 conceptual gaps remain underaddressed. First, immersive presentation is often implicitly treated as equivalent to improved execution [26,31], yet complex skill learning can be constrained by a cognitive-motor translation bottleneck: forming a representation of “what to do” does not necessarily ensure accurate embodied reproduction of “how to do it” [32]. Movement-encoding knowledge refers to the learner’s immediate cognitive representation, recall, and recognition of essential movement elements and sequence-relevant cues. Such encoding is a necessary early step in learning a novel movement sequence and can be assessed comparably across VR and video conditions without treating device-specific tracking metrics as equivalent to motor skill. This distinction is especially salient for Tai Chi, where successful learning requires not only recognizing key movement elements but also coordinating posture, timing, and spatial control. Second, the mechanisms through which VR may confer value are frequently reduced to broad notions of enjoyment or novelty [33,34], while state-level conditions that shape practice quality, such as perceived competence, pressure or tension, and affective experience during learning [15], are less systematically integrated into the evidence chain. We refer to these motivational and experiential states collectively as human factors outcomes. The enclosed nature of a head-mounted display may plausibly create a more private practice context by limiting external social cues, potentially reducing pressure and negative affect while supporting engagement [17,31,35]; however, these claims require disciplined testing alongside learning outcomes, rather than being inferred from immersion alone. At the same time, feasibility depends on clearly defined safety and tolerability boundaries because immersion-related symptoms can emerge even in well-controlled settings [29].

### Research Questions and Hypotheses

Guided by the identified gaps in older-adult immersive motor learning, we examined whether the more enclosed practice context created by a head-mounted display was associated with lower pressure or tension and more favorable competence-related and flow-related states. We posed the following research questions (RQs):

1. RQ1 (movement-encoding performance): When the instructional content is matched, does immersive VR produce higher posttest Tai Chi movement-encoding knowledge performance than standardized 2D video instruction after controlling for pretest score and age?
2. RQ2 (human factors outcomes): Does immersive VR systematically reshape older adults’ motivational and experiential states during training (eg, interest or enjoyment, perceived competence, pressure or tension, immersion, flow, and engagement) relative to standardized video instruction?
3. RQ3 (safety, tolerability, and feasibility): In real-world community deployment, what safety, tolerability, and feasibility profile does immersive VR show, including VR discomfort or symptoms, discontinuation or exclusion due to discomfort or technical failure, and the proportion of participants reporting zero Virtual Reality Sickness Questionnaire (VRSQ) symptoms?

Based on these questions, we specified the following hypotheses:

1. H1 (movement-encoding performance): Compared with the Video Group, the VR Group will show higher adjusted posttest movement-encoding knowledge scores when controlling for pretest performance and age.
2. H2 (human factors benefit): Compared with the Video Group, the VR Group will report a more favorable motivational and experiential profile, reflected in higher perceived competence and flow or immersion and lower pressure or tension.

Because safety, tolerability, and feasibility were evaluated descriptively, RQ3 was exploratory, and no directional hypothesis was specified.

In addition, for the VR Group, system logs (recognition accuracy, pose accuracy, and movement distance) were used to characterize performance descriptively during task execution, providing process-level signals to complement self-reports and learning outcomes.

## Methods

### Participants

A total of 106 community-dwelling older adults were recruited and enrolled between November and December 2025. Eligibility criteria were age ≥60 years, a native Chinese language background, and no self-reported major physical or mental disorders that could materially affect task participation or outcome assessment. The sample size was determined based on feasibility considerations and prior community-based studies with comparable designs. Participants were randomly assigned to either the VR Group (n=56) or the Video Group (n=50). Randomization was not stratified by age or prior Tai Chi experience; the observed age imbalance was, therefore, handled analytically by including age as a covariate and by conducting an exploratory Group × Age sensitivity analysis. Following quality control, 95 participants were retained as valid baseline cases for demographic and background comparisons (VR: n=46; Video: n=49).

### Experimental Design

We used a 2-arm pretest/posttest control-group design. The independent variable was learning modality (immersive VR learning vs 2D video-based learning). Sessions were conducted in quiet, spacious indoor settings coordinated by local community and village activity rooms. Before each session, the research team confirmed that participants had sufficient, unobstructed space for safe movement, and on-site supervision was provided throughout.

### Randomization and Allocation

The study leader generated an approximately 1:1 computer-based randomization sequence before study initiation. Allocation was implemented after eligibility screening, and recruitment and on-site coordination staff followed the prespecified allocation list for scheduling and documentation. The sequence was not stratified by age or prior Tai Chi experience. Participants completed the learning tasks under their assigned condition unless they withdrew or were excluded based on predefined invalid-sample criteria (eg, VR-related discomfort, technical failure, no usable data, or incomplete participation). Study-level exclusions were applied according to feasibility and data-quality criteria before the final analytic datasets were constructed. Participant flow, invalid cases, and exclusions from the primary analysis are reported in the *Results* section.

### Apparatus and Materials

The VR intervention was delivered using Meta Quest 3 and Meta Quest 3S head-mounted displays (Meta Platforms, Inc). Both models use the Snapdragon XR2 Gen 2 platform (Qualcomm Technologies, Inc) and provide comparable 6 df inside-out tracking with touch plus controllers, ensuring consistent tracking fidelity and interaction across devices. The Video Group learned the same content via standard 2D instructional videos presented on a conventional display.

### Procedure

All participants completed a pretest, the assigned learning session, and a posttest within the same study workflow. The learning content comprised 5 core Tai Chi forms delivered in a standardized order and format across conditions. After the learning session, participants completed postintervention questionnaires assessing intrinsic motivation, game-related experience, and engagement. For VR participants, the system additionally recorded behavioral performance indices during task execution (eg, recognition accuracy, pose accuracy, and movement distance) for descriptive reporting.

Training sessions were supervised on-site. Participants were instructed to stop immediately if they experienced dizziness, nausea, discomfort, or instability. Short breaks were allowed upon request, and the session was terminated if symptoms persisted. Any adverse events (eg, falls, near-falls, musculoskeletal pain, or persistent cybersickness symptoms) and reasons for discontinuation were documented by the staff. These procedures were implemented to minimize physical risks during standing practice among older adults and to ensure that cybersickness and balance-related safety were treated as part of real-world feasibility.

### Primary Outcome: Movement-Encoding Knowledge

The primary outcome was short-term movement-encoding knowledge, defined as immediate encoding, recall, and recognition of essential movement elements and sequence-relevant cues. It was assessed using a 5-item scoring rubric developed for the present study and guided by motor-learning frameworks [8,32]. The measure was administered before and after the learning session. It should be interpreted as a cognitive movement-representation outcome, not as a direct assessment of full Tai Chi motor execution, movement quality, or long-term skill acquisition [8,32]. Each item corresponded to one Tai Chi form and was scored from 0 to 4 using a predefined rubric (total range 0‐20; higher scores indicate more complete encoding and recall of movement elements). Total scores were computed only when all 5 items contained valid responses; otherwise, the total score was treated as missing for the primary analysis [36]. The 5-item scoring components are provided in Multimedia Appendix 1. Diagnostic reliability was acceptable for this rubric-based score (Cronbach α: pretest=0.910, posttest=0.725; McDonald’s omega total: pretest=0.914, posttest=0.743), although these coefficients should be interpreted cautiously because the measure was designed as a scoring rubric rather than a fully validated psychometric scale.

### Secondary Outcome: Intrinsic Motivation

Intrinsic motivation refers to participants’ immediate interest or enjoyment, perceived competence, and pressure or tension during the learning session. It was measured with 9 items from the Intrinsic Motivation Inventory (IMI) using a 1‐7 response scale: interest/enjoyment (5 items, including 1 reverse-scored item), perceived competence (2 items), and pressure/tension (2 items, including 1 reverse-scored item). Subscale scores were calculated as item means; higher scores indicate more of the named construct. Prior confirmatory work supports the IMI subscales’ factorial validity and internal consistency [37].

### Secondary Outcome: Game Experience

Game experience refers to participants’ immediate cognitive and affective experience of the learning environment. It was measured with the 14-item Game Experience Questionnaire (GEQ) core module using a 1 to 5 response scale. Seven 2-item subscales were calculated as the item means: competence, immersion, flow, tension, challenge, negative affect, and positive affect; higher scores indicate more of the named experience. Item allocation and scoring followed the GEQ manual [38]. Independent validation has raised concerns about the stability of the original 7-factor structure [39]; in the current sample, Cronbach α values for the seven 2-item subscales ranged from 0.462 to 0.728. GEQ subscale findings were therefore interpreted cautiously and as immediate experience indicators rather than definitive latent-trait measures.

### Secondary Outcome: Learning Engagement

Learning engagement refers to the vigor, dedication, and absorption that participants reported during the learning activity. It was measured with the 9-item Utrecht Work Engagement Scale–Short Form (UWES-S) using a 1 to 7 response scale. Vigor, dedication, and absorption scores were calculated as the means of 3 items each, and total engagement as the mean of all 9 items; higher scores indicate stronger engagement. Cross-national validation supports the scale’s 3-factor structure and internal consistency [40].

### Safety, Tolerability, and Feasibility

Safety and tolerability refer to VR-related symptoms and the ability to complete the session without clinically relevant discomfort or instability. VR symptoms were measured in the VR Group with the 9-item VRSQ (item scores of 0-3). Oculomotor and disorientation scores were normalized to 0 to 100 and averaged to obtain a total score; higher scores indicate more severe symptoms. We also reported the absolute number and proportion with zero symptoms. The VRSQ was developed with evidence of internal consistency and convergent validity for VR-specific symptoms [41]. Feasibility was defined descriptively through participant flow and the number and reasons for discontinuation or exclusion, including discomfort, technical failure/no usable data, and incomplete participation. It was not treated as a separate psychometric construct.

### Exploratory VR Behavioral Performance

VR behavioral performance refers to device-recorded process indicators available only in the VR Group. Recognition accuracy was the percentage of correct responses across 5 recognition items; pose accuracy was the percentage of correctly hit pose targets (15 total); and motion distance was the summed movement distance of the head and both hands in meters. These telemetry variables were summarized descriptively and explored using correlations; they were not designated primary or secondary learning outcomes.

### Data Quality Control and Analytic Samples

Participant flow and exclusions followed a 2-layer rule consistent with CONSORT-EHEALTH (Consolidated Standards of Reporting Trials of Electronic and Mobile Health Applications and Online Telehealth; Checklist 1) reporting guidance [42]. First, study-level invalid cases were removed based on predefined criteria (VR: n=10; Video: n=1), including motion sickness/discomfort, technical failure/no usable data, and incomplete participation/withdrawal, resulting in 95 valid baseline participants. Second, for the primary learning-outcome analysis, participants were included only if age, pretest score, and posttest score were all available, yielding the primary analytic sample of 92 (VR: n=45; Video: n=47). Missingness was limited: in the valid baseline sample, the Video Group had 2 out of 49 participants missing posttest scores, whereas the VR Group had 1 out of 46 missing pretest data and 1 out of 46 missing posttest data. All analyses used complete-case data for the variables involved.

### Statistical Analysis

All analyses were conducted in R software (v4.6.0; R Foundation for Statistical Computing). Descriptive statistics are reported as mean (SD) for continuous variables and n (%) for categorical variables. Baseline group differences were examined using independent-samples 2-tailed Welch *t* tests (continuous variables) and Fisher exact tests (categorical variables). The primary intervention effect on posttest performance was evaluated using analysis of covariance (ANCOVA) with posttest score as the dependent variable, group as the fixed factor, and pretest score and age as covariates; adjusted group differences are reported with 95% CIs and partial η^2^ [43,44]. For user-experience outcomes, group differences were examined using independent-samples 2-tailed Welch *t* tests with false discovery rate (FDR) adjustment applied across self-report outcomes [45,46]; Cohen *d* values were computed as VR minus Video. Given the baseline age imbalance, we additionally conducted age-adjusted linear models for key self-report outcomes and an exploratory Group × Age model for the primary outcome. Missing data were handled using complete-case analysis for the variables involved; a conservative sensitivity check implemented under the pretest-complete rule did not alter the primary conclusion. VR telemetry correlations with movement-encoding scores and selected self-report measures were examined descriptively using Pearson correlations.

### Ethical Considerations

The study protocol was reviewed and approved by the Research Office of Xi’an Translation University on October 16, 2025. The reviewing body did not issue a case or application number. All participants provided written informed consent before participation. Participants were informed of the study procedures and potential risks, including possible VR-related discomfort, such as dizziness or eye strain, and could rest, discontinue a session, or withdraw at any time without penalty. Study data were deidentified for analysis and reporting. Participants received a small commemorative gift valued at approximately RMB $40 to $50 (RMB $1=US $0.14 as of December 8, 2025) for each study visit.

### Trial Registration

This report presents first-session findings from a larger pilot randomized trial that was retrospectively registered with the International Traditional Medicine Clinical Trial Registry (registration number ITMCTR2026001760). The registry application was first submitted on April 1, 2026, after participant recruitment had commenced. The registry record includes additional follow-up assessments that are outside the scope of the present report.

## Results

### Participant Flow and Baseline Characteristics

As shown in Figure 1, a total of 106 older adults were assessed and randomized (VR: n=56; Video: n=50). Based on predefined invalid-sample criteria, 10 participants in the VR arm were excluded from the study (motion sickness/discomfort, n=3; technical failure/no usable data, n=3; incomplete, withdrew, or other, n=4), and 1 participant in the video arm was excluded (incomplete/withdrew, n=1), yielding 95 valid participants for baseline characterization (VR: n=46; Video: n=49), as shown in Table 1 (the 2 groups did not differ significantly in sex, education, or prior Tai Chi experience, but the Video Group was older on average). For the primary movement-encoding analysis, an additional 1 VR participant and 2 Video participants were excluded under the complete-case rule for age, pretest score, and posttest score, resulting in a final analytic sample of 92 participants (VR: n=45; Video: n=47), which was used for the ANCOVA reported in Table 2.

As shown in Table 1, baseline characteristics were summarized for the valid sample (N=95, Video Group n=49, VR Group n=46). Participants were older adults (overall mean age of 68.67, SD 7.55 y). The Video Group was older (mean 70.69, SD 7.43) than the VR Group (mean 66.52, SD 7.15 y), and this difference was statistically significant (*P*=.006). Sex distribution did not differ between groups (*P*=.16; overall: male 24/95, 25% and female 71/95, 75%). Educational attainment was also comparable across groups (*P*=.41), with the largest categories being primary school or below (31/95, 33%) and junior high school (28/95, 29%). Prior Tai Chi experience was similarly balanced (*P*=.77), with most participants reporting no prior exposure (72/95, 76%).

**Figure 1.**
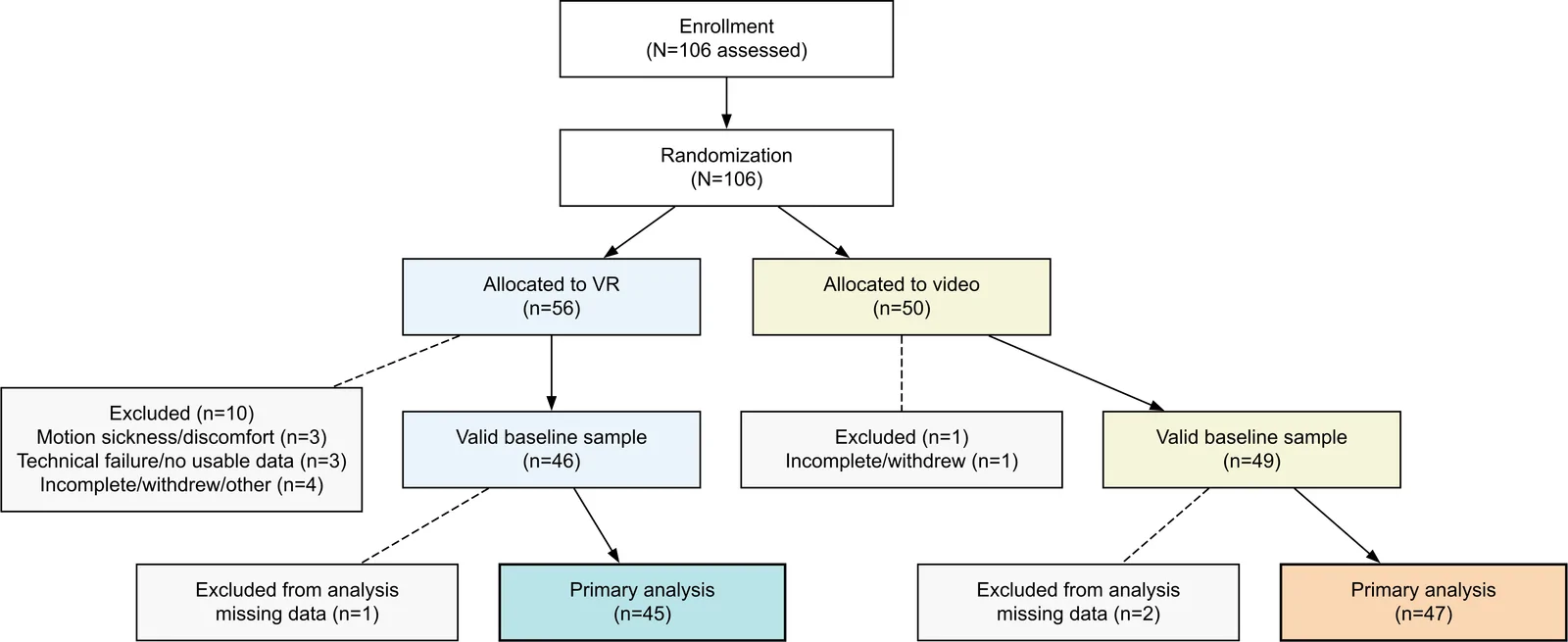
CONSORT (Consolidated Standards of Reporting Trials) flow diagram for the randomized comparison of immersive virtual reality (VR) and video-based Tai Chi movement-encoding instruction among community-dwelling older adults, November to December 2025. A total of 106 participants were assessed and randomized (VR Group: n=56; Video Group: n=50). Study-level exclusions were based on prespecified invalid-sample criteria (VR: n=10; Video: n=1), including motion sickness or discomfort (VR: n=3), technical failure or no usable data (VR: n=3), and incomplete participation, withdrawal or other (VR: n=4; Video: n=1). Valid participants contributing to baseline characteristics are reported in Table 1 (VR: n=46; Video: n=49). Primary outcome analyses used the complete-case primary sample (Table 2; VR: n=45; Video: n=47).

**Table 1.** Baseline characteristics of community-dwelling older adults randomized to immersive VR^a^ or video-based Tai Chi movement-encoding instruction, November to December 2025.^b^

Characteristic	Overall (N=95)	Video Group (n=49)	VR Group (n=46)	*P* value
Age (y), mean (SD)	68.67 (7.55)	70.69 (7.43)	66.52 (7.15)	.006
Sex, n (%)				.16
Male	24 (25)	9 (18)	15 (33)	
Female	71 (75)	40 (82)	31 (67)	
Education, n (%)				.41
Bachelor’s degree or above	4 (4)	2 (4)	2 (4)	
Middle school	28 (29)	13 (27)	15 (33)	
Junior college	6 (6)	1 (2.0)	5 (11)	
High school or vocational school	26 (27)	15 (31)	11 (24)	
Primary school or below	31 (33)	18 (37)	13 (28)	
Prior Tai Chi experience, n (%)				.77
Never	72 (76)	37 (76)	35 (76)	
Practice often	5 (5)	2 (4)	3 (7)	
Occasionally watched	9 (10)	6 (12)	3 (7)	
Practiced a little	9 (10)	4 (8)	5 (11)	

a VR: virtual reality.

b Values are mean (SD) for continuous variables and n (%) for categorical variables. Group differences were tested using independent-samples 2-tailed Welch *t* tests for age and Fisher’s exact tests for categorical variables. Missing data were not included in the summaries.

**Table 2.** Pretest-posttest Tai Chi movement-encoding knowledge scores and adjusted intervention effect in community-dwelling older adults randomized to immersive VR^a^ or video instruction, November to December 2025.^b^

Outcome	Video Group (n=47), mean (SD)		VR Group (n=45), mean (SD)		
Pretest score (range 0‐20)	2.70 (5.09)		2.80 (4.98)		
Posttest score (range 0‐20)	8.81 (4.97)		8.71 (4.49)		
Movement-encoding gain	6.11 (5.88)		5.91 (6.19)		

a VR: virtual reality.

b The adjusted mean difference between the VR and Video Groups was −0.269 (95% CI −2.25 to 1.72; *P*=.79), based on an analysis of covariance model with posttest score as the dependent variable, group as the fixed factor, and pretest score and age as covariates (primary analysis sample, N=92). Participants were included in the primary analysis only if age and all 5 pretest and posttest items were complete.

### Primary Outcome: Movement-Encoding Knowledge

As shown in Figure 2, total movement-encoding knowledge scores (0‐20) increased from pretest to posttest in both groups within the primary analysis sample (N=92), consistent with the descriptive results summarized in Table 2. The plotted means (SE) indicate comparable patterns of improvement across the Video Group (n=47) and the VR Group (n=45) under the same scoring rule and sample definition used for the main inferential model.

**Figure 2.**
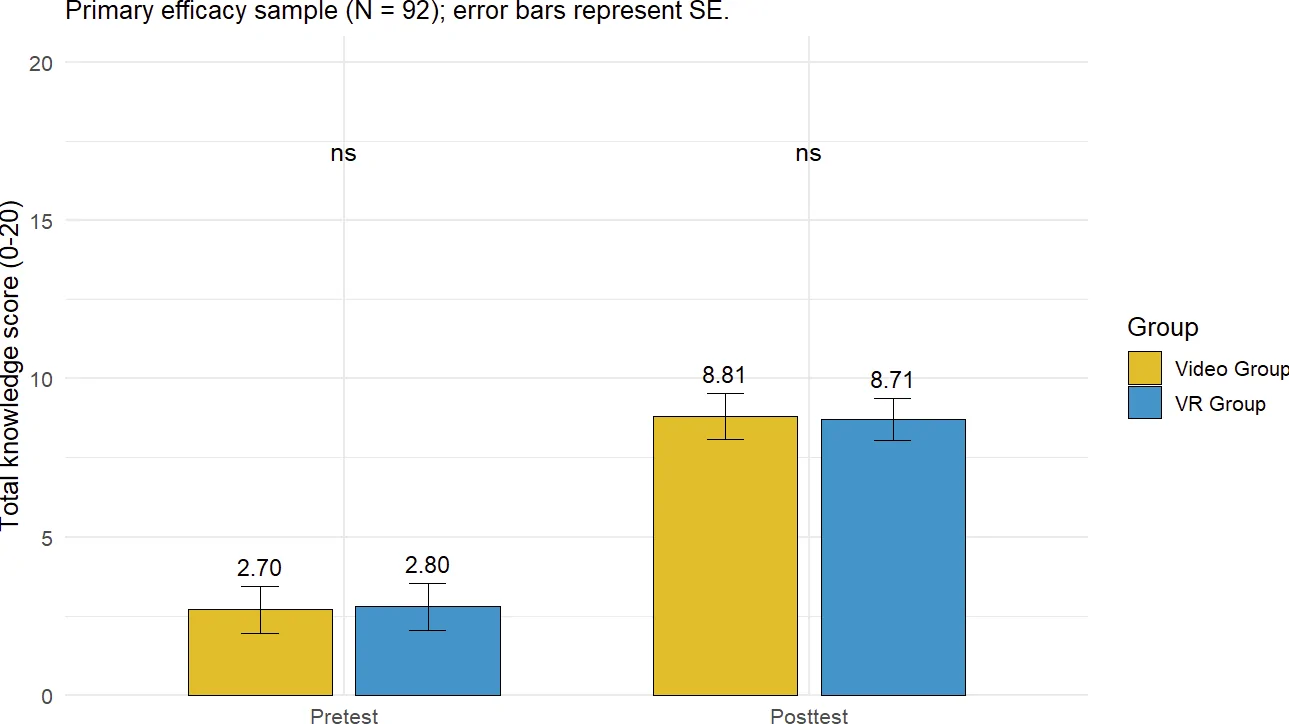
Pretest and posttest Tai Chi movement-encoding knowledge scores among community-dwelling older adults randomized to immersive virtual reality (VR) or video instruction, November to December 2025. Scores represent the sum of 5 movement-encoding knowledge items (each scored 0 to 4), yielding a total score range of 0 to 20. Bars show the mean; error bars represent the SE. Results are based on the primary analysis sample (primary efficacy sample: N=92; Video Group: n=47; VR Group: n=45) using the same scoring and inclusion rules as Table 2. ns: not statistically significant.

As shown in Table 2, the primary analysis sample included 92 participants (Video Group n=47, VR Group n=45) with complete pretest, posttest, and age data. Descriptively, pretest movement-encoding scores were comparable between groups (Video: mean 2.70, SD 5.09; VR: mean 2.80, SD 4.98), as were posttest scores (Video: mean 8.81, SD 4.97; VR: mean 8.71, SD 4.49) and movement-encoding gain (Video: mean 6.11, SD 5.88; VR: mean 5.91, SD 6.19). In the ANCOVA model controlling for pretest score and age, the adjusted intervention effect (VR vs Video) on posttest performance was not significant (adjusted difference −0.269, 95% CI –2.25 to 1.72; *P*=.79; partial η^2^=0.001).

### Secondary Outcome: Intrinsic Motivation

As shown in Table 3, intrinsic motivation outcomes were compared between the Video Group (n=47) and the VR Group (n=46) across 3 subscales. Mean scores were similar for interest or enjoyment (Video: mean 5.23, SD 1.33; VR: mean 5.53, SD 1.10), perceived competence (Video: mean 5.47, SD 1.87; VR: mean 5.86, SD 1.27), and pressure/tension (Video: mean 2.27, SD 1.36; VR: mean 2.39, SD 1.65). None of the between-group differences reached statistical significance, and all findings remained nonsignificant after FDR correction (*q*=0.376, *q*=0.376, and *q*=0.879, respectively).

**Table 3.** Intrinsic motivation after a single Tai Chi movement-encoding session in community-dwelling older adults randomized to immersive VR^a^ or video instruction, November to December 2025.^b^

IMI^c^ subscales (range 1-7)	Video Group (n=47), mean (SD)	VR Group (n=46), mean (SD)	*P* value	*q* value
Interest or enjoyment	5.23 (1.33)	5.53 (1.10)	.24	0.376
Perceived competence	5.47 (1.87)	5.86 (1.27)	.24	0.376
Pressure or tension	2.27 (1.36)	2.39 (1.65)	.69	0.879

a VR: virtual reality.

b Between-group differences were tested using independent-samples 2-tailed Welch *t* tests. False discovery rate adjustment was applied across the self-report outcome set to obtain *q* values.

c IMI: Intrinsic Motivation Inventory.

### Secondary Outcome: Game Experience

As shown in Figure 3, GEQ ratings showed the clearest experiential separation between groups. The figure displays the GEQ dimensions most relevant to the revised interpretation: competence, flow, tension, and challenge. FDR-adjusted *q* values are shown for visualization. VR participants reported higher competence and flow and lower tension after FDR correction, whereas the lower challenge rating in the VR Group was significant before correction but did not remain significant after FDR adjustment.

**Figure 3.**
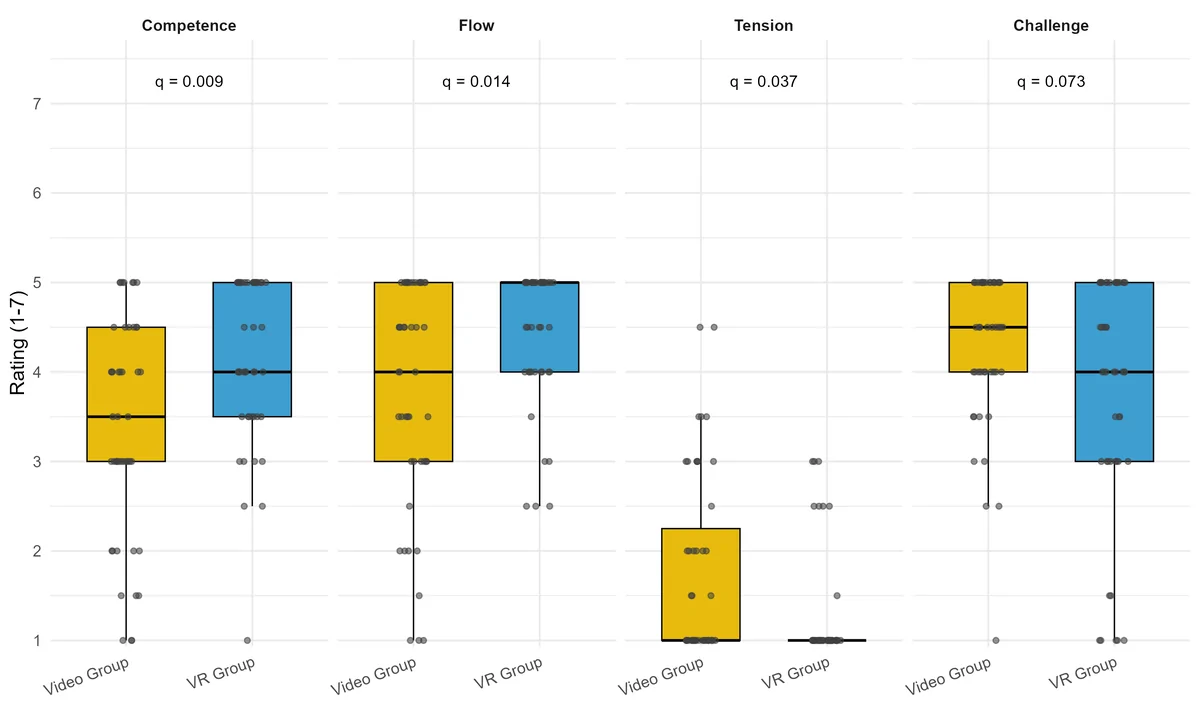
Game experience after a single Tai Chi movement-encoding session in community-dwelling older adults randomized to immersive virtual reality (VR) or video instruction, November to December 2025. Ratings are shown for Game Experience Questionnaire (GEQ) competence, flow, tension, and challenge. Box plots display the distribution of participant responses, with jittered points indicating individual observations. False discovery rate–adjusted *q* values are shown for the plotted GEQ subscales; inferential interpretation should follow the *q* values reported in Table 4.

**Table 4.** Game experience after a single Tai Chi movement-encoding session in community-dwelling older adults randomized to immersive VR^a^ or video instruction, November to December 2025.^b^

GEQ^c^ dimensions (range 1-5)	Video Group (n=47), mean (SD)	VR Group (n=46), mean (SD)	*P* value	*q* value
Competence	3.36 (1.21)	4.15 (0.92)	<.001	0.009
Immersion	4.10 (0.95)	4.37 (0.77)	.13	0.302
Flow	3.77 (1.26)	4.46 (0.76)	.002	0.014
Tension	1.76 (1.04)	1.27 (0.63)	.008	0.037
Challenge	4.29 (0.86)	3.74 (1.33)	.02	0.073
Negative affect	1.83 (1.04)	1.83 (1.08)	.99	0.987
Positive affect	4.24 (0.87)	4.49 (0.77)	.16	0.310

a VR: virtual reality.

b Between-group differences were tested using independent-samples 2-tailed Welch *t* tests. False discovery rate adjustment was applied across the self-report outcome set to obtain *q* values.

c GEQ: Game Experience Questionnaire.

As shown in Table 4, game experience (GEQ; 1‐5) differed on several dimensions between the Video Group (n=47) and the VR Group (n=46). The VR Group reported higher competence (Video: mean 3.36, SD 1.21; VR: mean 4.15, SD 0.92; *P*<.001, *q*=0.009; Cohen *d*=0.733) and higher flow (Video: mean 3.77, SD 1.26; VR: mean 4.46, SD 0.76; *P*=.002, *q*=0.014; Cohen *d*=0.661) after FDR correction. The VR Group also reported lower tension (Video: mean 1.76, SD 1.04; VR: mean 1.27, SD 0.63; *P*=.008, *q*=0.037; Cohen *d*=−0.562). Challenge was lower in the VR Group before correction (Video: mean 4.29, SD 0.86; VR: mean 3.74, SD 1.33; *P*=.02; Cohen *d*=−0.491), but this comparison did not remain significant after FDR adjustment (*q*=0.073). No significant between-group differences were observed for immersion, negative affect, or positive affect after correction.

### Secondary Outcome: Learning Engagement

As shown in Table 5, learning engagement (UWES-S; 1‐7) was broadly comparable between the Video Group (n=47) and the VR Group (n=46). Total engagement did not differ significantly (Video: mean 5.48, SD 1.49; VR: mean 5.70, SD 1.43; *q*=0.669), nor did the vigor (Video: mean 5.68, SD 1.43; VR: mean 5.65, SD 1.67; *q*=0.987) or dedication subscales (Video: mean 5.50, SD 1.54; VR: mean 5.55, SD 1.57; *q*=0.987). Absorption showed a modest, nonsignificant trend favoring the VR Group (Video: mean 5.26, SD 1.85; VR: mean 5.89, SD 1.42; *P*=.07), which remained nonsignificant after FDR adjustment (*q*=0.192).

**Table 5.** Learning engagement after a single Tai Chi movement-encoding session in community-dwelling older adults randomized to immersive VR^a^ or video instruction, November to December 2025.^b^

UWES-S^c^ dimensions (range 1-7)	Video Group (n=47), mean (SD)	VR Group (n=46), mean (SD)	*P* value	*q* value
Total engagement	5.48 (1.49)	5.70 (1.43)	.48	0.669
Vigor	5.68 (1.43)	5.65 (1.67)	.93	0.987
Dedication	5.50 (1.54)	5.55 (1.57)	.89	0.987
Absorption	5.26 (1.85)	5.89 (1.42)	.07	0.192

a VR: virtual reality.

b Between-group differences were tested using independent-samples 2-tailed Welch *t* tests. False discovery rate adjustment was applied across the self-report outcome set to obtain *q* values.

c UWES-S: Utrecht Work Engagement Scale–Short Form.

### Exploratory VR Behavioral Performance

As shown in Table 6, the VR Group’s recognition accuracy averaged 75.22% (SD 31.04%), pose accuracy averaged 41.16% (SD 25.15%), and total motion distance was averaged 245.87 (SD 80.23) meters. Among participants with complete paired data (n=44), recognition accuracy was positively associated with posttest movement-encoding knowledge (*r*=0.317, *P*=.04), whereas pose accuracy was not (*r*=0.129, *P*=.41). These device-recorded indicators are exploratory process measures and should not be interpreted as direct tests of Tai Chi motor execution.

**Table 6.** Exploratory device-recorded behavioral performance during immersive virtual reality Tai Chi movement-encoding instruction among community-dwelling older adults, November to December 2025.^a^

Metric	Mean (SD)
Recognition accuracy (%)	75.22 (31.04)
Pose accuracy (%)	41.16 (25.15)
Motion distance (m)	245.87 (80.23)

a Recognition accuracy is the percentage of correct responses across 5 recognition items. Pose accuracy is the percentage of correctly hit pose targets (15 total). Motion distance is the sum of movement distances across the head and both hands (m). Correlations with posttest movement-encoding knowledge used 44 complete paired observations.

### Safety, Tolerability, and Feasibility

Safety and tolerability outcomes are shown in Table 7. Among VR participants with valid data (n=46), the mean VRSQ total score was 3.77 (SD 11.12) on a 0 to 100 scale; oculomotor and disorientation scores were 2.90 (SD 9.50) and 4.64 (SD 13.25), respectively. A total of 36 out of 46 (78%) participants reported zero symptoms. Among participants with complete paired data (n=45), VRSQ total scores were positively associated with GEQ tension (*r*=.458, *P*=.002). Feasibility was reflected in participant flow: of 56 participants assigned to VR, 46 contributed valid baseline data; study-level exclusions included motion sickness or discomfort (n=3), technical failure or no usable data (n=3), and incomplete participation, withdrawal, or other reasons (n=4).

**Table 7.** Safety, tolerability, and feasibility of a single immersive virtual reality Tai Chi movement-encoding session among community-dwelling older adults, November to December 2025.^a^

Metric	Values
VRSQ^b^ total (range 0‐100), mean (SD)	3.77 (11.12)
Oculomotor	2.90 (9.50)
Disorientation	4.64 (13.25)
Zero symptom rate, n/N (%)	36/46 (78)

a Virtual Reality Sickness ﻿Questionnaire (VRSQ) total and subscale scores are scaled from 0 to 100, with higher values indicating more severe symptoms. The zero symptom rate is the absolute number and percentage of virtual reality participants with a total VRSQ symptom sum of 0. The correlation between VRSQ total scores and Game Experience Questionnaire (GEQ) tension scores was based on 45 complete paired observations. Feasibility exclusions are reported in Figure 1 because they occurred before the construction of the valid baseline sample.

b VRSQ: Virtual Reality Sickness ﻿Questionnaire.

## Discussion

### Key Findings and Contribution

This randomized study found no evidence that immersive VR improved short-term Tai Chi movement-encoding knowledge relative to content-matched video instruction. After accounting for baseline knowledge and the between-group age difference, posttest performance did not differ between conditions. Among participants who completed the VR session, however, game-related competence and flow were higher, and tension was lower. In this setting, the main observed difference between the delivery modes was, therefore, experiential rather than cognitive performance.

### Movement-Encoding Knowledge

Richer sensory presentation does not necessarily produce better learning [19,25,47]. In this study, scores increased descriptively from pretest to posttest in both groups, but VR did not yield higher adjusted posttest performance. This result is consistent with the view that learning gains depend strongly on instructional method and practice structure rather than on the delivery medium alone [19,48]. In Tai Chi, visual representation must also be converted into embodied execution; without haptic guidance, expert correction, or repeated practice, immersive visualization alone may be insufficient to outperform a carefully designed video after a single session [8,48]. Recent evidence indicates that motor benefits are more likely to emerge in repeated, adaptive, or higher-dose VR training than in a single exposure [21-23]. The null between-group result means that the experiential findings should be interpreted separately from claims of improved learning [15,19,32].

### Game Experience

The clearest between-group differences were in game experience. VR participants reported higher competence and flow and lower tension, while negative affect was comparable across conditions. These results do not support the assumption that older adults necessarily experience head-mounted displays as more stressful or intimidating [49]. One possible explanation is that VR created a more private, less socially evaluative practice context by reducing external distractions and limiting visible comparisons with others [16,50,51]. This interpretation remains speculative because the study did not directly measure perceived privacy, social-evaluative threat, self-consciousness, or cognitive load. The headset-based setting may have supported concentration and perceived control, but that mechanism requires direct testing [15,16,52].

### Intrinsic Motivation and Learning Engagement

Notably, the advantages observed in the game experience did not extend to intrinsic motivation, general engagement, or posttest movement-encoding performance. This dissociation is theoretically informative. Higher perceived competence in VR may reflect a stronger immediate sense of control or confidence in the practice environment, but that feeling did not translate into superior movement-encoding scores within a single session. It is also possible that the immersive presentation induced overconfidence, or that the outcome measure captured cognitive representation more than actual execution quality. Measures such as flow, tension, and perceived competence are sensitive to immediate task conditions, whereas stable motivational orientations, generalized engagement, and measurable motor learning may require repeated exposure, autonomy-supportive scaffolding, and longer time horizons to shift [52-54].

Importantly, differences between GEQ and IMI on conceptually similar dimensions (eg, tension or pressure) may reflect item-level nuance rather than contradiction [37,38,55]. GEQ tension items tend to capture situational frustration or irritation during interaction, whereas IMI pressure or tension items more directly reflect performance-related anxiety or feeling pressured to do well [38]. In this study, immersive VR may have reduced interaction-related tension without materially changing performance-evaluative pressure, which could remain salient in a single-session learning context [54,56]. This dissociation suggests VR operates more as an interface and interaction buffer than a direct remedy for performance anxiety, highlighting design opportunities (eg, supportive feedback or guided practice) to target the latter [16,53,57].

### Safety, Tolerability, and Feasibility

The safety profile was encouraging but not uniform. Simulator-sickness symptoms were generally low among VR participants with valid data, and most completers reported no symptoms. At the same time, attrition related to motion sickness and technical problems was not trivial and forms part of the intervention’s feasibility and tolerability profile [24,28-30,49]. These exclusions show that acceptability varies across individuals and hardware-software circumstances [24,28-30,49]. Symptom estimates reflect participants who remained in the protocol; therefore, safety conclusions should acknowledge possible survivorship bias and the need for structured screening and monitoring in real-world use [24,28,30,49]. The favorable experiential profile among VR completers may not generalize to older adults who are more susceptible to motion sickness or device-related discomfort.

### Practical Implications for Community Implementation

Because the intervention was delivered in community and village activity rooms, the study provides initial evidence of supervised use outside a laboratory [17,24,28,58,59]. For short-term movement-encoding knowledge, no between-group difference was detected; the distinctive findings for VR concerned competence, flow, and tension [16,19,34]. The study did not measure long-term adherence, so implications for sustained participation remain untested. Three practical safeguards are suggested by the feasibility results: (1) brief prescreening for motion sensitivity and comfort with head-mounted displays, (2) gradual exposure with short initial bouts, and (3) reliable on-site support for headset fit, tracking, and interruptions [28,49,58]. VR telemetry may also inform future feedback design and training adjustment [34,58], but these process measures are not interchangeable with learning outcomes. Finally, VR differed from video in immersion, interactivity, embodiment, and sensorimotor coupling; the present design cannot isolate those components.

### Limitations and Threats to Interpretation

Several limitations shape interpretation. First, the primary outcome was a brief movement-encoding knowledge test that emphasizes cognitive representation, recall, and recognition of movement elements more than objective movement quality; it cannot establish actual Tai Chi execution, long-term retention, transfer to real-world practice, or expert-rated motor competence [8,32,49]. Second, the analysis focused on immediate posttest performance; comparative benefits of VR may emerge more clearly in delayed retention, transfer, or adherence over time [8,28,49]. Third, the primary analysis required complete pretest and posttest data, while VR-specific attrition due to sickness and technical failure constrains generalizability and should be addressed as an outcome in its own right [28,49]. Fourth, randomization was not stratified by age or prior Tai Chi experience, and the Video Group was older at baseline. Age was controlled in the primary analysis, and exploratory analyses offered no evidence that age modified the intervention effect; however, future trials should consider stratified randomization or minimization procedures.

### Future Directions

Future research should move beyond binary comparisons toward mechanism-driven and heterogeneity-driven designs [58,60]. A priority is to test the proposed immersive-shielding pathway by directly measuring social-evaluative threat, perceived privacy, self-consciousness, cognitive load, and self-efficacy as potential mediators linking condition to tension and flow [10,16,50]. Studies should add objective motor metrics (expert ratings, kinematic measures, or pose estimation) to determine whether experiential advantages translate into cleaner movement execution or safer performance under balance demands [16,32]. Longer follow-up is needed to evaluate retention, transfer, and adherence, domains where reduced tension and higher perceived competence may prove consequential but were not tested here [8,15,16]. Finally, safety should be modeled proactively: rather than treating cybersickness as incidental, future trials should identify predictors of intolerance and establish screening and adaptive onboarding protocols so that immersive training can be targeted to those most likely to benefit while minimizing risk for those vulnerable to adverse symptoms [28,49,58].

### Conclusions

In this randomized study, immersive VR did not produce higher short-term Tai Chi movement-encoding knowledge than standardized video instruction after adjustment for baseline performance and age [61,62]. Among participants who completed the VR session, perceived competence and flow were higher and tension was lower, suggesting a more favorable immediate game-experience profile [31,62]. These experiential findings do not establish superior learning or motor execution. Discomfort-related withdrawals and technical exclusions also place clear limits on feasibility and generalizability [63-65]. Overall, VR may be useful when experiential engagement is a priority, but the present results do not show an advantage over video for immediate movement-encoding performance.

## Supplementary material

10.2196/96867 Multimedia Appendix 1 Five-item movement-encoding scoring rubric for the Tai Chi knowledge test.

10.2196/96867 Checklist 1 CONSORT-EHEALTH checklist (V1.6).

## Abbreviations

ANCOVA: analysis of covariance
CONSORT-EHEALTH: Consolidated Standards of Reporting Trials of Electronic and Mobile Health Applications and Online Telehealth
FDR: false discovery rate
GEQ: Game Experience Questionnaire
IMI: Intrinsic Motivation Inventory
RQ: research question
UWES-S: Utrecht Work Engagement Scale–Short Form
VR: virtual reality
VRSQ: Virtual Reality Sickness Questionnaire
