# Supplementary material for: Knowledge Performance, User Experience, and Cybersickness With Immersive Virtual Reality for Tai Chi Movement-Encoding in Older Adults: Randomized Comparison Study
Source: JMIR Aging. 2026 Sep 30;9:e96867. doi: 10.2196/96867 (PMC13626395; doi:10.2196/96867)
Supplement: Supplementary file 1 [file aging-v9-e96867-s002.pdf]

CONSORT-EHEALTH V1.6 Checklist

Prepared for JMIR Aging manuscript #96867: Immersive VR for Tai Chi Movement-Encoding in Older Adults: A Randomized Comparison of Knowledge Performance, User Experience, and Cybersickness

This completed Word checklist follows CONSORT-EHEALTH V1.6.1 and accompanies the final revised manuscript for JMIR Aging manuscript #96867.

Items marked 'Partly addressed' identify reporting limits that are stated transparently in the checklist and manuscript.

CONSORT-EHEALTH item	EHEALTH extension / checklist focus	Location in revised manuscript	Completed response / notes for submission
1a. Title	Identify randomized design and eHealth mode of delivery; identify the target group.	Title	Addressed. The title identifies immersive virtual reality, Tai Chi movement-encoding, older adults, and randomized comparison. The title also specifies knowledge performance, user experience, and cybersickness as major outcomes.
1b. Structured abstract	Describe key intervention/comparator components, recruitment/assessment mode, human involvement, use/uptake data, and negative-trial interpretation where relevant.	Abstract	Addressed. The structured abstract reports N=106 randomized, the primary ANCOVA result, key GEQ findings, VR telemetry, VRSQ symptoms, and the trial registration (ITMCTR, ITMCTR2026001760; retrospectively registered).
2a. Scientific background and rationale	Describe the problem, system/solution, comparator rationale, and stakeholder context.	Introduction	Addressed. The manuscript frames Tai Chi learning in older adults, access barriers to standardized instruction, immersive VR as a possible scalable training environment, and the need for a strong content-matched 2D video comparator.
2b. Specific objectives or hypotheses	No eHealth-specific addition.	Research Questions and Hypotheses	Addressed. The manuscript states research questions on movement-encoding knowledge, motivational and experiential outcomes, and feasibility/safety, followed by hypotheses.
3a. Trial design	No eHealth-specific addition.	Methods - Experimental Design; Trial Registration	Addressed. The study is described as a two-arm pretest-posttest randomized comparison of immersive VR versus content-matched 2D video instruction. The present report covers first-session findings from a larger pilot randomized trial.
3b. Important changes after trial commencement	Report bug fixes, downtimes, content changes, staff changes, system failures, and other unexpected events.	Methods - Randomization and Allocation; Results - Participant Flow	Partly addressed. The manuscript reports technical failure/no usable data as a study-level exclusion reason in the VR arm. No major protocol changes, content changes, or planned intervention updates are reported in the manuscript.
4a. Eligibility criteria	Clarify computer/Internet literacy if relevant, recruitment information, and whether recruitment/assessment was online or face-to-face.	Methods - Participants; Ethics Approval	Addressed. Eligibility was age >=60 years, native Chinese language background, and no self-reported major physical or mental disorders materially affecting participation. Participants were recruited offline from community/village settings, not through open online self-enrolment. Written informed consent was obtained.
4b. Settings and data-collection locations	Clarify whether outcomes were self-assessed through online questionnaires or otherwise.	Methods - Experimental Design; Measures; Ethics Approval	Addressed. Sessions were conducted face-to-face in quiet, spacious indoor community and village activity-room settings. Outcomes included administered pre/post movement-encoding tests, self-report questionnaires, and VR system telemetry; the study was not an online-only trial.
5-i. Developers, sponsors, and owners	Mention names, credentials, affiliations of developers/sponsors/owners and declare any relation to the system.	Methods - Apparatus and Materials; Declarations	Partly addressed. The manuscript describes the VR apparatus and intervention, states that no financial support was received, and includes a Conflicts of Interest statement ('None declared'). Detailed software ownership and developer credentials are not separately reported.
5-ii. Development history and formative evaluation	Describe application history, development process, and previous usability/formative evaluations if applicable.	Methods - Apparatus and Materials	Partly addressed. The manuscript describes the study intervention and materials. No prior formal formative evaluation is reported.
5-iii. Revisions/updating/version	Report intervention/comparator version/date and whether content was frozen during the evaluation.	Methods - Apparatus and Materials; Interventions	Partly addressed. The manuscript reports the evaluated hardware (Meta Quest 3/3S) and standardized instructional content. A

			precise software version number is not available in the study record.
5-iv. Quality assurance	Describe quality assurance methods for information accuracy and quality.	Methods - Apparatus and Materials; Measures	Addressed in part. The intervention used standardized Tai Chi instructional content and a content-matched 2D video comparator. The five-item movement-encoding rubric is provided as Multimedia Appendix 1.
5-v. Replicability materials	Provide screenshots, screen-capture video, flowcharts, source code, or similar replication materials where possible.	Methods; Figures; Multimedia Appendix	Partly addressed. The manuscript includes Figure 1 for participant flow and Multimedia Appendix 1 for the scoring rubric. Screenshots or screen-capture examples of the VR and video learning environments are not included.
5-vi. Digital preservation and URL	Provide URL or preservation route for the evaluated application where applicable.	Not applicable / not publicly hosted	Not applicable or not publicly accessible. The intervention was delivered in supervised offline/community sessions using headset-based VR rather than an open-access web application. No public URL is reported.
5-vii. Access	Describe how participants accessed the system, setting/context, payment, membership, and platform/Internet access.	Methods - Experimental Design; Apparatus and Materials	Addressed. Participants accessed the VR system or video instruction during supervised study sessions in community/village indoor settings. They did not need to pay for the system or independently access an online platform.
5-viii. Mode of delivery, features, components, and theoretical framework	Describe delivery mode, features/functionality, intervention content, comparator, tailoring, feedback, progress tracking, communication channels, and instructional strategy.	Abstract; Introduction; Methods - Apparatus and Materials; Interventions	Addressed. The intervention was head-mounted immersive VR for Tai Chi movement-encoding; the comparator was standardized content-matched 2D video instruction. The instructional comparison focused on matched Tai Chi content with different delivery modes, not therapist-guided telehealth or asynchronous communication.
5-ix. Use parameters / dose	Describe intended dose, timing, frequency, and instructions to users.	Methods - Experimental Design; Interventions	Addressed in part. The study used a single-session pre-test/training/post-test design. Participants completed the learning task under their assigned condition during the study session. No long-term repeated-use dose was tested.
5-x. Human involvement and technical assistance	Clarify human involvement and technical assistance in the intervention and as trial support.	Methods - Experimental Design; Ethics Approval	Addressed. On-site research staff coordinated sessions, confirmed safe movement space, supervised participation, and allowed rest/discontinuation. The intervention was not an unsupervised home-based eHealth intervention.
5-xi. Prompts/reminders	Report prompts/reminders used to use the application.	Not applicable	Not applicable. The study used supervised single-session exposure; no emails, SMS, app notifications, or longitudinal use reminders were reported.
5-xii. Co-interventions, training, and support	Describe additional interventions, training sessions, or support.	Methods - Experimental Design; Ethics Approval	Addressed. Both arms received standardized learning exposure to the same Tai Chi content through the assigned modality. On-site supervision and safety support were provided during participation; no additional therapeutic co-intervention is reported.
6a. Outcomes	Define primary/secondary outcomes, online questionnaire validation if applicable, use/dosage monitoring, and qualitative feedback if any.	Methods - Primary and Secondary Outcome subsections; Statistical Analysis; Multimedia Appendix 1	Addressed. The primary outcome was a five-item movement-encoding knowledge score (0-20) assessed pre/post. Secondary outcomes are separated into intrinsic motivation (IMI), game experience (GEQ), learning engagement (UWES-S), and safety/tolerability (VRSQ and participant flow). VR telemetry is explicitly exploratory. Scoring, interpretation, and psychometric support are reported; the rubric is provided in Multimedia Appendix 1.
6b. Outcome changes after trial commencement	No eHealth-specific addition.	Methods / Results	No outcome changes are reported in the manuscript.
7a. Sample size	Describe whether expected attrition was considered.	Methods - Participants	Addressed. The manuscript states that sample size was determined based on feasibility considerations and prior community-based studies with comparable designs. Attrition/exclusion is transparently reported in Figure 1 and Results.
7b. Interim analyses / stopping guidelines	No eHealth-specific addition.	Not applicable	Not applicable. No interim analyses or early stopping guidelines are reported for this single-session community study.
8a. Random sequence generation	No eHealth-specific addition.	Methods - Randomization and Allocation	Addressed. The study leader generated an approximately 1:1 computer-based randomization sequence before study initiation.
8b. Type of randomization	No eHealth-specific addition.	Methods - Participants; Randomization and Allocation	Addressed. Participants were randomly assigned approximately 1:1 to VR or video. Randomization was not stratified by age or prior Tai Chi experience.
9. Allocation concealment	No eHealth-specific addition.	Methods - Randomization and	Partly addressed. Allocation was implemented after eligibility screening using a pre-specified

mechanism		Allocation	allocation list. The manuscript does not describe sealed envelopes, centralized allocation, or another formal concealment mechanism.
10. Implementation	Who generated the sequence, enrolled participants, and assigned interventions.	Methods - Randomization and Allocation	Addressed. The study leader generated the computer-based randomization sequence. After eligibility screening, recruitment and on-site coordination staff followed the prespecified allocation list for assignment, scheduling, and documentation.
11a. Blinding	Specify who was and was not blinded; acknowledge if participant blinding was impossible.	Methods / Discussion - Limitations	Partly addressed. Because VR and 2D video are visibly different modalities, participant blinding was not feasible. The manuscript does not report blinded outcome assessors or blinded data analysts.
11b. Similarity of interventions	Usually refers to placebo/sham similarity; describe comparator similarity where relevant.	Methods - Interventions	Addressed. The comparator was an active, content-matched 2D video instruction condition. The study intentionally varied delivery mode while matching Tai Chi instructional content.
12a. Statistical methods	Specify handling of attrition/missing data and how non-use/dropout was treated.	Methods - Statistical Analysis	Addressed. The primary ANCOVA used post-test score as outcome and group, pre-test score, and age as predictors. Missing data were handled by complete-case analysis for variables involved, and a conservative sensitivity check under the pre-test-complete rule did not alter the primary conclusion.
12b. Additional analyses	No eHealth-specific addition.	Methods - Statistical Analysis; Results	Addressed. The manuscript reports FDR-adjusted self-report comparisons, age-adjusted sensitivity analyses, an exploratory Group x Age model, VR telemetry correlations, and missing-data checks.
X26. Ethics and informed consent	Report ethics approval, consent procedures, safety/privacy procedures, and harm detection/reduction.	Methods - Ethics Approval; Declarations	Addressed. The protocol was reviewed and approved on October 16, 2025; written informed consent was obtained; participants were informed of VR-related discomfort risks, could rest or discontinue at any time, and study data were anonymized.
13a. Participant flow	Report numbers randomly assigned, received intended intervention, and analyzed; flow diagram recommended.	Figure 1; Results - Participant Flow and Baseline Characteristics	Addressed. Figure 1 and Results report 106 randomized (VR n=56; Video n=50), valid baseline cases (VR n=46; Video n=49), and primary analysis sample (VR n=45; Video n=47; N=92).
13b. Losses and exclusions	Report attrition/exclusions and, if possible, usage/dose/engagement figures.	Figure 1; Results - Participant Flow and Baseline Characteristics	Addressed. VR exclusions were motion sickness/discomfort (n=3), technical failure/no usable data (n=3), incomplete/withdrew/other (n=4); Video exclusion was incomplete/withdrew (n=1). Primary analysis excluded additional missing pre/post data (VR n=1, Video n=2 as reported in the manuscript).
14a. Recruitment and follow-up dates	Report recruitment/follow-up periods and secular events if relevant.	Methods - Participants; Figure 1	Addressed. Participants were recruited and the reported first-session procedures were conducted during November-December 2025. The registry record includes additional follow-up assessments outside the scope of this report.
14b. Why trial ended or stopped	No eHealth-specific addition.	Methods / Results	Not applicable. The manuscript does not report early stopping; the study appears to have ended after completing planned feasible community recruitment/sessions.
15. Baseline data	Report demographics relevant to digital divide, such as age, education, gender, socioeconomic status, and eHealth literacy if known.	Table 1; Results - Participant Flow and Baseline Characteristics	Addressed. Table 1 reports baseline demographic/background characteristics including age, gender, education, and prior Tai Chi experience. Age imbalance is acknowledged and addressed analytically.
16. Numbers analyzed	Report multiple denominators and define use/exposure thresholds; ITT preferred, secondary user analyses need caveats.	Abstract; Figure 1; Results	Addressed with caveat. The manuscript reports multiple denominators: randomized N=106, valid baseline N=95, and primary complete-case analysis N=92. The primary analysis is complete-case rather than strict ITT, and the manuscript explains missing data and sensitivity checks.
17a. Outcomes and estimation	Report primary/secondary outcomes, process outcomes, use/intensity metrics, effect sizes, and precision.	Abstract; Results; Tables 2-7	Addressed. Results report the adjusted primary effect with 95% CI, P value, and partial eta squared; separate IMI, GEQ, and UWES-S results; exploratory VR telemetry in Table 6; and safety/tolerability in Table 7.
17b. Binary outcomes	Report both absolute and relative effects where relevant.	Results; Table 7	Addressed where applicable. The VRSQ zero-symptom count is reported as 36/46 (78%). Exclusion counts and reasons are reported by group in Figure 1 and the Results.
18. Ancillary analyses	Describe subgroup/exploratory analyses and distinguish pre-specified from exploratory; user-only analyses need caveats.	Methods - Statistical Analysis; Results	Addressed. Ancillary analyses include FDR correction, age-adjusted sensitivity checks, a Group x Age exploration, VR telemetry correlations, psychometric diagnostics, and missing-data checks. VR-only telemetry analyses are interpreted descriptively.

19. Harms and unintended effects	Include privacy breaches, technical problems, qualitative feedback/observations, and unintended effects.	Results; Discussion; Ethics Approval	Addressed. VR discomfort/symptoms were measured using VRSQ; 3/56 VR participants were excluded for motion sickness/discomfort and 3/56 for technical failure/no usable data. Data anonymization and the option to rest/discontinue are reported.
20. Limitations	Discuss eHealth-specific limitations, lack of blinding, multiplicity, non-use/usability issues, consent bias, and unexpected events.	Discussion - Limitations	Addressed. The manuscript discusses movement-encoding versus motor execution, short-term assessment, lack of long-term retention/adherence data, survivor/complete-case limitations among VR completers, technical exclusions, cybersickness, age imbalance, and inability to separate immersion from interactivity/embodiment.
21. Generalizability	Discuss applicability beyond RCT context, routine implementation differences, training/support, and population differences.	Discussion - Limitations and Practical Implications	Addressed. Generalizability is limited to community-dwelling older adults able to tolerate supervised VR exposure. The manuscript cautions that experiential benefits are mainly among VR completers and may not generalize to all older adults assigned to VR or to unsupervised home use.
22. Interpretation	Restate study questions, summarize answers from primary and process outcomes, and highlight future research.	Discussion - Key Findings and Contribution; Conclusions	Addressed. The discussion interprets the null primary movement-encoding result alongside favorable GEQ outcomes and low symptoms among valid VR completers, while calling for objective motor assessment, delayed follow-up, and longer-term adherence research.
23. Registration	Registration number and registry name.	Abstract; Methods - Trial Registration	Addressed. The larger pilot randomized trial was retrospectively registered with the International Traditional Medicine Clinical Trial Registry (ITMCTR; registration number ITMCTR2026001760). The registry application was first submitted on April 1, 2026, after participant recruitment had commenced.
24. Protocol	Where the full trial protocol can be accessed, if available.	Methods - Trial Registration	Partly addressed. A protocol was submitted as part of the ITMCTR registration record. A public protocol URL is not reported in the manuscript.
25. Funding	Sources of funding and role of funders.	Declarations - Funding	Addressed. The manuscript states that the authors received no financial support for the research, authorship, and/or publication of the article.
X27. Competing interests	State financial/nonfinancial interests and the relation of the study team to the evaluated system.	Declarations - Conflicts of Interest	Addressed. The manuscript includes a Conflicts of Interest subsection stating 'None declared.'
