# Supplementary material for: Knowledge Performance, User Experience, and Cybersickness With Immersive Virtual Reality for Tai Chi Movement-Encoding in Older Adults: Randomized Comparison Study
Source: JMIR Aging. 2026 Sep 30;9:e96867. doi: 10.2196/96867 (PMC13626395; doi:10.2196/96867)
Supplement: Supplementary file 2 [file aging-v9-e96867-s001.docx]

**Multimedia Appendix 1**

**Five-item movement-encoding scoring rubric for the Tai Chi knowledge test**

# Purpose and construct

This appendix describes the five-item movement-encoding knowledge score used as the primary learning outcome in the manuscript. The score was designed to assess immediate encoding, recognition, and recall of Tai Chi movement-name associations and key movement elements. It should not be interpreted as an expert-rated assessment of full Tai Chi motor execution, movement quality, long-term retention, or real-world transfer.

# Target Tai Chi forms

The pre-test and post-test each contained five items corresponding to the same five target Tai Chi forms, presented in a fixed content set across study conditions.

| Item | Chinese name | English descriptive label |
| --- | --- | --- |
| 1 | 野马分鬃 | Parting the Wild Horse's Mane |
| 2 | 白鹤亮翅 | White Crane Spreads Its Wings |
| 3 | 双峰贯耳 | Double Peaks Strike the Ears |
| 4 | 闪通臂 | Flash Through the Arms |
| 5 | 搬拦捶 | Deflect, Parry, and Punch |

# Test structure

The analytic dataset contained five pre-test item scores (Pretest1-Pretest5) and five post-test item scores (Posttest1-Posttest5). Each item was coded on a 0-4 rubric. The total pre-test and post-test scores were calculated by summing the five item scores, yielding a total range of 0-20 at each time point.

The broader experimental task used a learning-recognition-recall sequence. Recognition tasks assessed the movement-to-name association, whereas recall tasks assessed the name-to-movement representation with spatial cue support. The five-item knowledge score summarizes the participant's movement-encoding performance for manuscript analysis and is distinct from the VR telemetry variables reported separately in Table 6, such as recognition accuracy, pose accuracy, and movement distance.

# Item-level scoring rubric

Each target movement item was scored from 0 to 4. Higher scores indicate more complete encoding of the target movement-name association and key movement elements.

| Score | Rubric interpretation |
| --- | --- |
| 0 | No recognizable encoding of the target movement-name association or key movement elements. |
| 1 | Minimal encoding; isolated cues or partial recognition were present, but the response was largely incorrect or incomplete. |
| 2 | Partial encoding; the participant showed some correct recognition or recall of the target movement, with substantial omissions or inaccuracies. |
| 3 | Mostly correct encoding; the participant recognized or recalled most key movement elements, with only minor omissions or inaccuracies. |
| 4 | Complete and accurate encoding; the participant correctly represented the target movement-name association and key movement elements. |

# Total score calculation

| Component | Calculation rule |
| --- | --- |
| Pre-test total score | Pretest1 + Pretest2 + Pretest3 + Pretest4 + Pretest5; range 0-20. |
| Post-test total score | Posttest1 + Posttest2 + Posttest3 + Posttest4 + Posttest5; range 0-20. |
| Movement-encoding gain | Post-test total score minus pre-test total score. |
| Missing-data rule | A total score was computed only when all five item scores at that time point were valid. If any of the five item scores was missing or invalid, the corresponding total score was treated as missing for the primary analysis. |

# Reliability diagnostics

Internal-consistency diagnostics were calculated for the five-item score in the primary analysis sample. These diagnostics support the coherence of the item set but should be interpreted cautiously because the measure is a task-specific scoring rubric rather than a fully validated psychometric scale.

| Diagnostic | Pre-test | Post-test |
| --- | --- | --- |
| Cronbach alpha | 0.910 | 0.725 |
| McDonald's omega total | 0.914 | 0.743 |

# Interpretive limitations

The movement-encoding score captures short-term cognitive representation of Tai Chi movement elements. It does not establish objective movement quality, expert-rated Tai Chi proficiency, delayed retention, adherence, balance transfer, or real-world motor competence. Accordingly, the main manuscript interprets the primary outcome as movement-encoding knowledge rather than direct motor execution.
